# Assessment of Microstructure and Mechanical Properties of Stir Zone Seam of Friction Stir Welded Magnesium AZ31B through Nano-SiC

**DOI:** 10.3390/ma12071044

**Published:** 2019-03-29

**Authors:** Vijayakumar Subramani, Balaji Jayavel, Ramesh Sengottuvelu, Prince Jeya Lal Lazar

**Affiliations:** 1 Department of Mechanical Engineering, University College of Engineering Kancheepuram, Kancheepuram 631552, India; svijayme@yahoo.co.in; 2 Department of Mechanical Engineering, Jayalakshmi Institute of Technology, Dharmapuri 636352, India; 3 Department of Mechanical Engineering, KCG college of Technology, Chennai 600097, India; ramesh_1968in@yahoo.com (R.S.); prince.mech@kcgcollege.com (P.J.L.L.)

**Keywords:** AZ31B magnesium alloy, SiC nano-particles, friction stir welding, microstructure, mechanical properties

## Abstract

In the present study, silicon carbide nanoparticles were incorporated into AZ31B magnesium alloy welded joints using the friction stir welding technique at five different stir zone volume fractions. The volume percentage of nano-SiC was varied from 0–20% in increments of 4%. Initially, the microstructure analyses of the V4, V8, and V12 welded joints were observed to be in good accordance with a homogeneous dispersion of nano SiC particles within the stir zone (SZ). Moreover, the particle’s agglomeration and large cluster size were found in the SZ due to insufficient heat generation of the specimen’s V16 and V20 during friction stir welding (FSW). Furthermore, the tensile and microhardness test was conducted, and the results indicate that the volume fractions increase along with the ultimate tensile strength and average microhardness, which increases up to 12% SiC addition (V12). With this effect, the fracture morphology was examined in the nano-composite joints that revealed a brittle fracture mode, which was observed in specimens V16 and V20, and the remaining was in the ductile fracture mode. From this investigation, a significant enhancement was found in the weld nugget zone that the tensile strength value of the V12 specimen was improved by 21% compared to the welded joint without SiC.

## 1. Introduction

Fuel economy is an important issue in automotive industries due to the high weight on the body structures [1,2]. To overcome this problem, a suitable alternative is needed; there is particular need for replacements for steel and aluminum structures that would not affect the properties of the product. In regard to replacing heavy materials with light materials, magnesium is a good substitute in automobile industrial structural works compared to aluminum because of its low density (1740 Kg/m^3^) and high strength to weight ratio [3]. Among the magnesium alloys, AZ31B wrought magnesium alloy has gained significant attention because it is the lightest structural metallic material with good stiffness, heat conductivity, excellent weldability/machinability, and higher corrosion/oxidation resistance [4]. Structural works were made by joining materials together under variable loads, and special attention has been given to the joining of magnesium alloys as an essential part of industries. In recent decades, friction stir welding (FSW) has offered many potential benefits for joining magnesium alloys in structural works. In 1991, the Welding Institute, UK invented the FSW process specifically for the development of aluminum and magnesium alloys. It is a solid-state welding technique used to recast the adjacent base plates along the weld joint line without melting the base material [5]. In recent decades, more attention has been paid by researchers to developing magnesium matrix composite (MMC) welded joints, which are obtained through the FSW process by adding ceramic reinforcements to the abutting edges of the plates. Ceramic particles are a proper driving force for producing high strength magnesium matrix composite joints because of the integration ability of the molten matrix, hard nature, and grain refining effect [6,7]. To achieve high strength magnesium matrix composite joints, conventional welding technique problems, such as porosity, hot cracking, and formation of surface oxide layers, have to be eliminated [8,9]. On the other hand, the critical issue of interfacial chemical reactions between the particles and the matrix alloy has been considered, which leads to the production of intermetallic components and alloy segregations that show a dominant effect on the mechanical properties [10,11].

Initially, Sun and Fujii et al. fabricated Cu/SiC composite welded joints obtained through the FSW process and observed that the SiC reinforced welded joint was completed in the copper surface without any defect. They also reported that a considerable amount of nugget zone strength was improved [12]. Accordingly, Mahmoud Abbasi et al. examined the effect of the SiC reinforcement on the microstructure, mechanical, and corrosion properties of the AZ31B/SiC welded joints. They observed that friction stir (FS) welded and processed joints showed better mechanical and corrosion properties than FS welded joints [13]. More recently, friction stir processing (FSP), an outcome of FSW, has emerged as a promising technique used to synthesise nano- and micro-sized reinforcements into the matrix alloy and fabricate surface composites [14]. By adopting this technique, the AZ31B/Al_2_O_3_ nano composite layer was fabricated, and it was reported that the multiple FSP passes and various tool pin profiles significantly influenced the distribution of the Al_2_O_3_ particles. Also, the columnar threaded tool pin profile improved the average microhardness value and reduced the cluster size [15]. The TiC particles were synthesised into AA6082 aluminum alloy with five different stir zone volume fractions, and the effect of the volume fractions on the microstructure and mechanical properties was analyzed. As a result, the variable volume fractions of the TiC particles reinforced processed plates significantly influenced the area of the surface composite, ultimate tensile strength, and average microhardness values [16]. Sathishkumar et al. produced Cu/B_4_C surface composites, varying the stir zone volume fraction on the microstructure and hardness fabricated by the FSP technique. They reported that when the volume fraction of the B_4_C particles increased, the microhardness value also increased from lower to higher volumes. An increased volume fraction influenced the area of the surface composite [17]. Moreover, a few researchers have paid attention to the study of the effect of the stir zone of various volume fractions on the microstructure and mechanical properties of FS processed plates.

From the above survey, it is evident that no author has thus far reported the friction stir welding of AZ31B magnesium alloy with SiC nanosized ceramic reinforcement incorporated along the weld joint line with five different stir zone volume fractions. In the present analysis, the deliberate effect of the SiC particle’s distributions and stir zone volume fraction on the microstructure and mechanical properties of magnesium matrix composite joints is established.

## 2. Materials and Methods 

**Figure 1:**
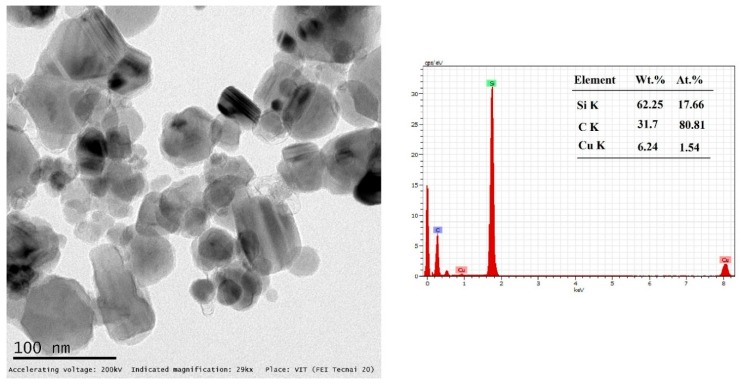
TEM micrograph with energy-dispersive X-ray spectroscopy (EDS) analysis of as-received SiC nanoparticles.

**Figure 2:**
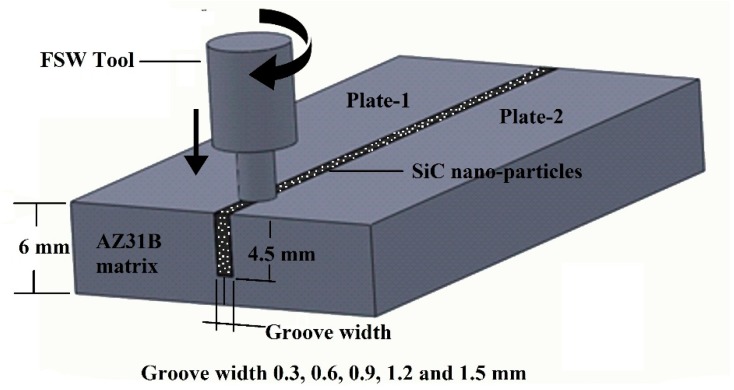
Schematic representation of the volume fraction sample.

In this experimentation, the FSW process was done using commercially available AZ31B magnesium alloy 6 mm thick plates (supplied by Luoyang Magnesium Gurnee Metal Material Co., Ltd., Luoyang, China). The chemical compositions and mechanical properties of the received matrix alloy are represented in Table 1 and Table 2. Figure 1 shows the TEM (FEI Tecnai 20, Hillsboro, OR, USA) micrographs of the as-received SiC nanoparticles (supplied by Hongwu International Group Ltd., Guangzhou, China) of the average particle size of 50 nm for the energy-dispersive X-ray spectroscopy (EDS, FEI Tecnai 20, Hillsboro, OR, USA) analysis. The dimensions of the weld plates were 75 mm width and 100 mm length; they were extracted from the base plate through the wire-cut electrical discharge machining (EDM) process. The width of the grooves were 0.3, 0.6, 0.9, 1.2, and 1.5 mm, and the corresponding volume percentages were represented in V4, V8, V12, V16, and V20, respectively. In each groove, width and depth were made in the weld joint line through the EDM process. Figure 2 is the schematic representation of volume fraction work piece with clear dimensions. 

**Figure 3:**
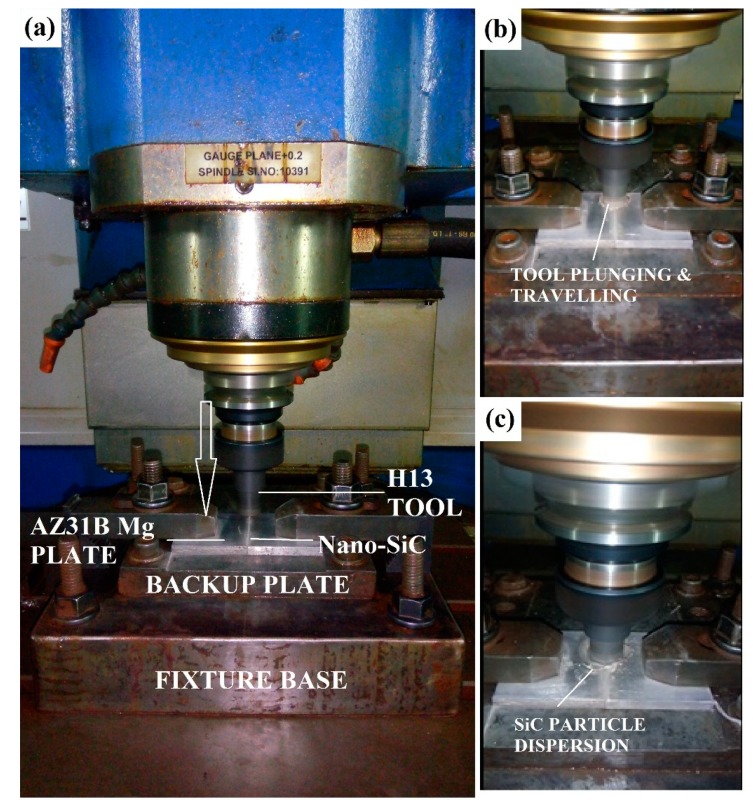
(**a**) FSW process setup with fixture base assembly (**b**) tool plunging and traveling (**c**) Nano-SiC particles dispersion.

Before the welding process, the SiC nanoparticles were compacted into the groove that was located in the weld center line of the adjoining plates. The FSW process was employed by using a computer numerical controlled vertical milling center (VMC) as a welding machine (machine supplied by ACE Manufactring Systems Ltd., Bengalure, India), and we developed a special fixture base to clamp the specimens, as shown in Figure 3. Initially, several trial experiments were conducted to produce AZ31B/SiC welded joints, and from the result of these trial experiments, process parameters were finalized as a constant tool rotational speed of 1250 rpm and a tool travel rate of 25 mm/min. The remaining FSW parameters selected in the study are listed in Table 3.

**Figure 4:**
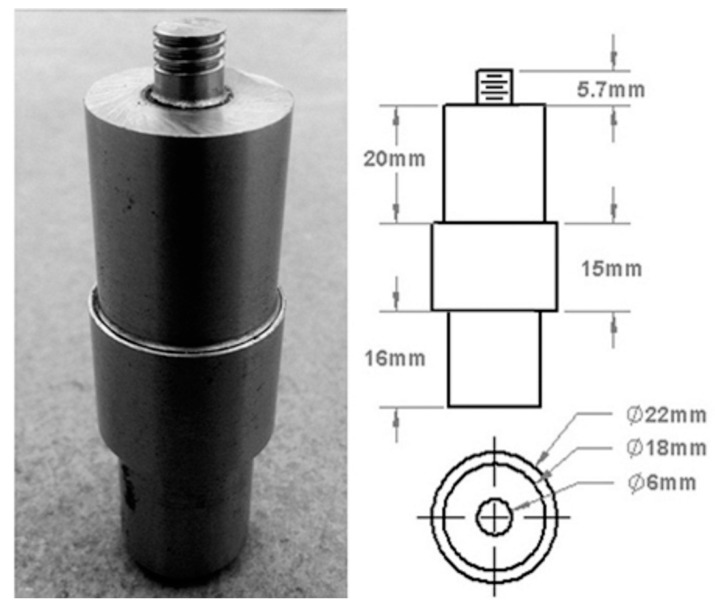
Pictorial representation of the H13 cylindrical threaded tool pin profile.

The prepared joints were taken to conduct cross-sectional examinations, and the results showed that good weld joints free from defects such as pinhole, tunnel defect, and kissing bonds could be produced irrespective of the volume fractions. The H13 hot-working tool steel with 56 HRC with a cylindrical threaded tool pin profile was used in this experimentation. Figure 4 shows the pictorial representation of the FSW tool with clear dimensions. According to the process parameters, five levels of volume fractioned AZ31B/SiC composite welded joints from V4 to V20 were developed. The theoretical and actual volume fractions of the SiC particles were determined using the following expressions.

V_Theoretical %_ = [A_groove_ /PA_tool pin_] × 100 (1)

V_Actual %_ = [A_groove_/A_surface composite_] × 100 (2)

A_groove_ = W_groove_ × D_groove_ (3)

PA_tool pin_ = D_Pin_ × L_Pin_ (4)

where V_Theoretical %_ and V_Actual %_ are the theoretical and actual volume fractions of the SiC particles, respectively; A_groove_ is the area of the groove; PA_tool pin_ is the projected area of the tool pin; A_surface-composite_ is the area of the surface composite; W_groove_ is the width of the groove; D_groove_ is the depth of the groove; D_Pin_ is the diameter of the pin; and L_pin_ is the length of the pin. 

**Figure 5:**
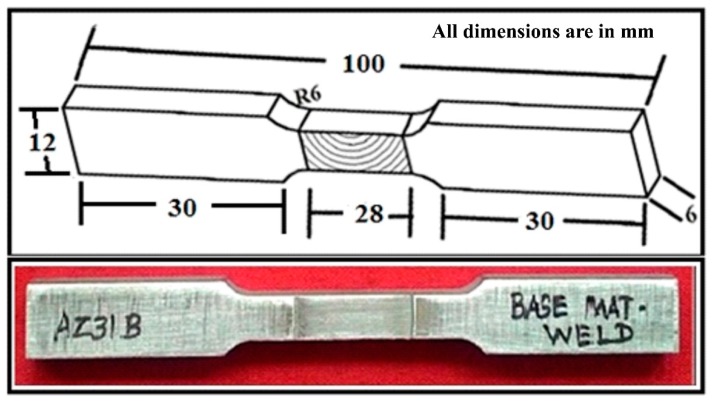
Schematic diagram of the tensile specimen with dimensions.

Metallographic examination of the welded joints was studied as per ASTM/E3-95 standard [15], and the specimens were prepared accordingly. Wire cut machined out samples were initially polished and etched with solutions of 10 mL acetic acid, 5 g picric acid, 10 mL water, and 100 mL ethanol. After etching, optical microscopy and SEM tests were conducted to find out the particle distributions, agglomerations, and particle-rich and particle-free fields on the cross-section of the weld nugget zone. As per ASTM-E8 standard [18], the tensile test specimen was extracted from the AZ31B/SiC composite welded joints normal to the welding direction using the wire cut EDM processes, as shown in Figure 5. The electronically controlled universal tensile testing machine was utilized to evaluate the ultimate tensile strength and percentage of elongation. The microhardness value was measured using Vicker’s microhardness tester (HMV-2T, SHIMADZU, Tokyo, Japan) to evaluate the average microhardness of the stir zone under the load of 25 g applied at 15 s. Microhardness surveys were taken at 1 mm constant distance at every ball indentation.

## 3. Results and Discussion

### 3.1. Macrostructure Observation

**Figure 6:**
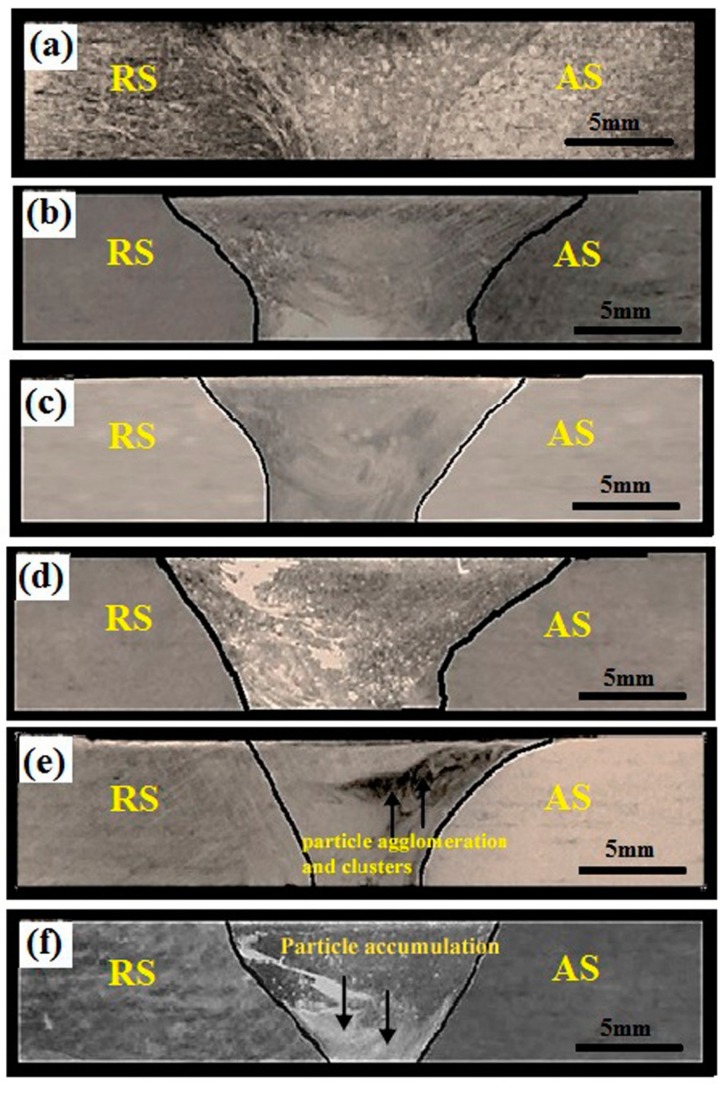
Macrostructure of the AZ31B/SiC welded joints and base metal weld joint without SiC particles (**a**) V0; (**b**) V4; (**c**) V8; (**d**) V12; (**e**) V16; (**f**) V20.

Figure 6 illustrates the five different volume fractions of the AZ31B/SiC welded joint macrostructure along with the base metal welded joint. Because of the tool rotation speed, traveling speed, tool material, tool pin profile, and heat absorption capacity of the matrix, a basin-shaped nugget zone was formed in all specimens [19,20]. The absence of identified defects such as tunnel defect, pinhole, kissing bond, and piping defect in specimen numbers V4 to V20 were noted [20]. However, in the case of samples V16 and V20 with severe SiC particles, accumulations were observed in the weld nugget zones, which were attributed to two important phenomena—the non-deformable SiC particles that acted as barriers to the free flow of plasticized AZ31B magnesium with an increase in the volume fraction of the stir zone, and the non-homogeneous distribution of SiC particles in the stir zone due to the lack of sufficient plasticization of the matrix material, which decreased during FSW while increasing the number of reinforcement particles achieved by increasing the groove size, which would not produce satisfactory martial flow around the tool pin [16,17]. From the specimens V16 and V20, it could be inferred that the nominal width of the groove was achieved in the cases of the V4, V8, and V12 samples. From the specimens V4, V8, and V12, it was concluded that the particles’ distributions around the pin were satisfactory, and there was enough plasticization of the matrix material that produced defect-free composite joints in the given welding conditions, as shown in Figure 6a–c.

**Figure 7:**
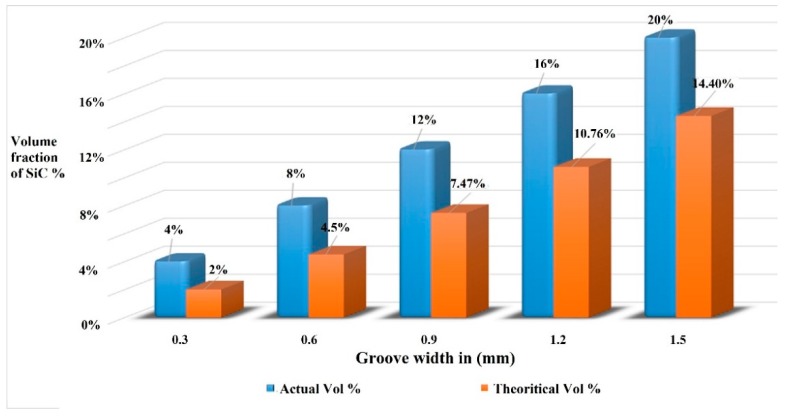
Comparison of the theoretical and actual volume fraction of SiC particles in the composite joints.

Variable stir zone volume fractions directly influenced the area of the friction stir welded zone that involved the SiC surface composite. The area of the surface composite (ASC) was measured using image analyzing software. The ASC depended on the amount of plasticization of the matrix material within the weld nugget zone. On the other hand, the factors responsible for the plasticization of the matrix material were the tool rotation speed and the area of groove width. The frictional heat generation increased in the nugget zone due to the increasing rotational speed, which improved the amount of plasticization of the molten matrix [21]. On the other hand, an increase in the stir zone (SZ) volume fraction with constant welding speed and feed condition decreased the amount of plasticization of the molten matrix within the SZ due to the lack of heat generation [16,17]. As a result, the ASC was an absolutely irreversible factor in the groove width of the weld plate. The SZ volume fraction of SiC increased from V4 to V20, which was achieved by varying the groove width from 0.3 to 1.5 mm, which was found to decrease the ASC from 67.41 mm^2^ to 46.85 mm^2^. Hence, the reduction of ASC while increasing the SZ volume fraction may have been due to any of the following reasons: (a) the lack of plasticization of the matrix in the stir zone, (b) the generation of flow stress within the SZ around the pin tool profile, which was offered by the non-deformable SiC particles, or (c) further increments of nano SiC leading to the reduction of heat generation in the SZ. During FSW, the same tool was utilized irrespective of the groove size to accommodate various volume fractions of the SiC particles. Hence, the theoretical ASC was equal to the projected area of the tool pin in all cases. The decrease in the ASC led to a drop in the actual volume fraction of the SiC particles in the surface composite. Figure 7 illustrates the comparison between the theoretical and actual volume fraction graphs. It could be inferred that more molten matrix was produced during the FSW process compared to the calculated theoretical molten matrix.

### 3.2. Microstructural Observations

**Figure 8:**
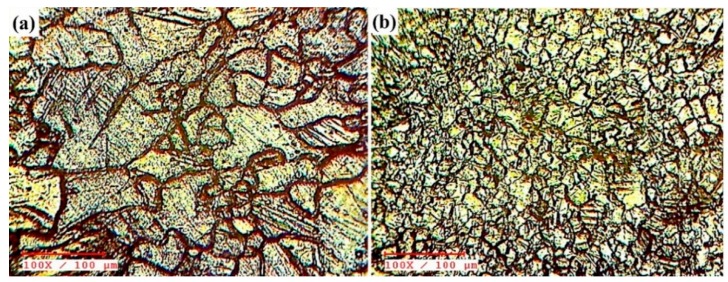
Optical microstructure: (**a**) as-received matrix alloy (**b**) FSW AZ31B joint (without reinforcement).

The effective combination of the tool rotational speed and tool traveling feed was an important parameter for the up-gradation of the fine equiaxed grain structure and enhancement of the particle’s distributions in the weld nugget zone during the FSW process [12,13]. Figure 8 shows the optical microstructure of the received matrix alloy (Figure 8a) and base metal welded joint without nano-SiC (Figure 8b). A received matrix alloy with distinguished grain boundary layers and FSW AZ31B joint was obtained from an SZ with fine equiaxed grains. There was clear that the high tool rotational speed led to vigorous stirring action that produced more frictional heat generation within the SZ and coarsened the grains [6,16,17,22].

**Figure 9:**
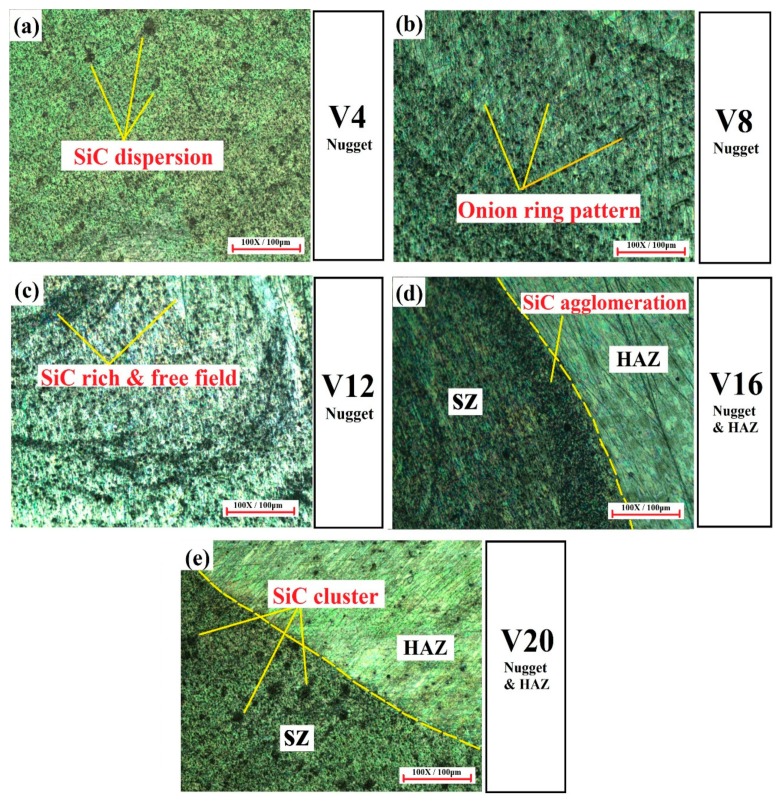
Optical micrographs of AZ31B/SiC composite welded joints. (**a**) V4; (**b**) V8; (**c**) V12; (**d**) V16; (**e**) V20.

Figure 9 shows the optical micrographs of the various volume fractioned AZ31B/SiC composite joints containing SiC obtained from the SZ and the heat affected zone (HAZ) with 100× magnification. When increasing the SZ volume fraction, the dispersion of SiC nanoparticles in the SZ was an important issue during the FSW process. The nano-sized SiC reinforcement particles were successfully distributed in the V4, V8, and V12 magnesium matrix, as shown in Figure 9a–c, whereas the dispersion of SiC particles was non-uniform in the V16 and V20 specimens due to particle agglomeration, as shown in Figure 9d,e. Two responsible factors led to the production of good particle distribution in the SZ—the dynamic recrystallization and the pinning effect of the SiC nanoparticles [23]. Due to the high rotational speed and lower traveling rate of the welding process, the material underwent severe plastic deformation at peak temperature, which produced more heat within the SZ, and the molten matrix was coarsened to form fine grains. This phenomenon is known as dynamic recrystallization [24]. Additionally, nano-sized SiC particles created nucleating sites in the SZ, which broke the initial grains and produced more low-angle, disoriented grain boundaries during recrystallization. This effect is known as the pinning effect [7,18]. The grain boundary refinement could be attributed to the formation of a complete onion ring pattern in the SZ, irrespective of the volume fractions. This caused a material flow pattern along with nano-SiC particles from the warmer zone in the top to the cooler zone below as a result of the pin driven flow [25]. Several investigators observed an onion ring structure, particle-rich, and particle-free fields during FSW and processing [6,7,15,18]. The onion ring pattern was found in the tapered pin tool profile during the FSW of AA7075/SiC joints [18] and also observed during the FSP of AZ31B/Al_2_O_3_ within the SZ [15].

The regular onion ring structure was obtained from the specimens of V8 and V12, as shown in Figure 9b,c. Generally, with variable SZ volume fractions and constant tool pin diameter, the amount of plasticization of the molten matrix in the SZ was an important phenomenon for producing good particle distributions and avoiding cluster formations during the FSW process [16,17,21]. The amount of matrix material reduced from V4 to V12 was achieved by improving the width of the groove as well as increasing the reinforcement that led to distributing the SiC particles within the SZ, as shown in Figure 9a–c. It was further evidenced that continuous dark thick lines indicating the reinforcement flow (particle-rich field) and continuous thin lines indicating molten matrix material flow (particle-free regions) were formed alternatively. Thus, it could be inferred that the specimens from V4 to V12 received sufficient heat input around the tool pin profile from the advancing side to the retracting side during the FSW process. [7,12,18]. On the other hand, an incomplete dispersion of nano SiC pattern was obtained between the nugget zone and the HAZ of specimens V16 and V20, as shown in Figure 9d,e. From these samples, it can be seen that the dispersion of nano SiC particles was not clear about the molten matrix, causing agglomeration and clusters in the advancing side of the weld nugget zone. The same effect was observed in the macroscopic examination of specimens V16 and V20 (Figure 6). However, the amount of reinforcement increased and reduced the spacing between the reinforcement particles when the SZ volume fraction was increased. Moreover, the reduction of space between the SiC particles led to a reduction of the temperature in the weld nugget zone [17,21]. Thus, it could be inferred that the V16 and V20 specimens produced insufficient heat during FSW due to inadequate stirring and material flow around the tool pin profile, which led to the formation of clusters in the nugget zone. 

**Figure 10:**
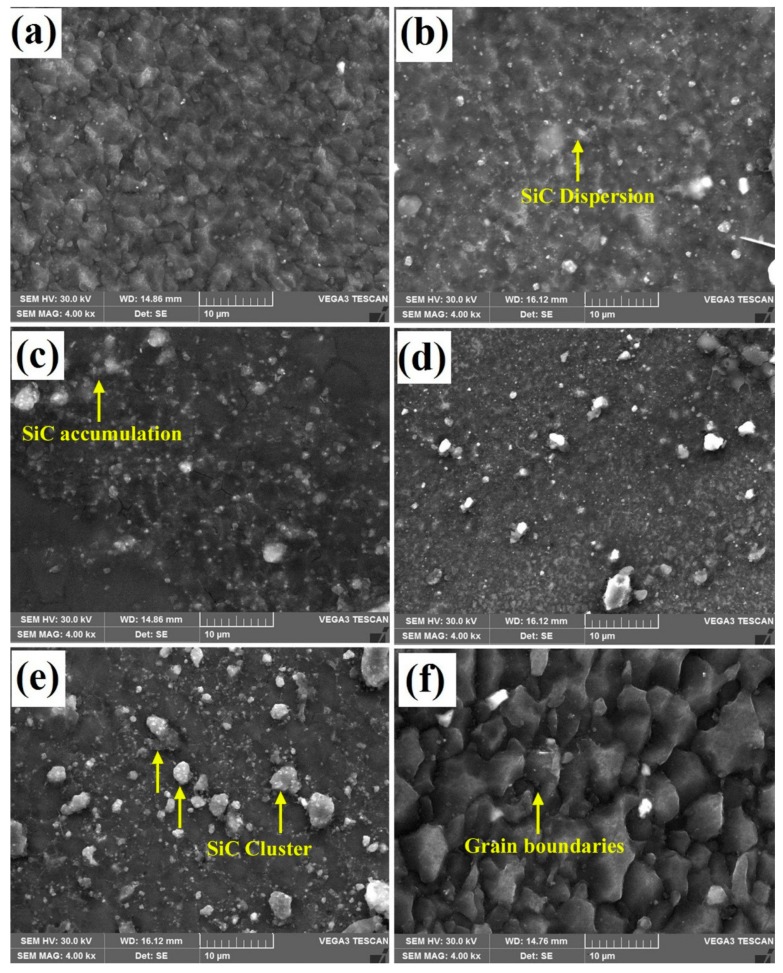
SEM micrograph of the AZ31B/SiC composite joints with fine distribution. (**a**) V4; (**b**) V8; (**c**) V12; (**d**) V16; (**e**) V20; (**f**) V0-FS welded without SiC.

**Figure 11:**
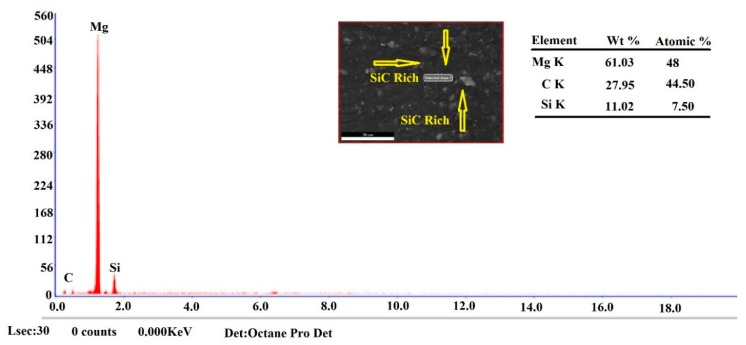
EDS peak points of reinforcement.

Figure 10 illustrates the SEM micrographs of the AZ31B/SiC nano composite joints with fine distribution of SiC particles and FS welded without SiC joint. It was evident that the improvisation of particles within the SZ (irrespective of volume fractions) led to the improvement of the SiC particle’s density in the grain structure. According to these figures, there was no interface between the SiC particles, and the magnesium matrix appeared to be clean and was not surrounded by any voids or reaction products. Several factors, such as constant rotational speed and feed, improved volume fractions, and inadequate heat generations, influenced the formations of the clusters in the SZ. Remarkably bigger sized clusters were observed in the specimens V16 and V20 compared with the other volume specimens. The prominent reason was the inadequate material mixing with the SiC particles due to lack of heat generation [15]. Moreover, the dispersion of particles was a significant issue of various volume fractioned welded joints that could have been enhanced by means of introducing more speed and feed during FSW, which will be the next study of the authors. The EDS peak points of the AZ31B/SiC reinforced composite welded joints are represented in Figure 11. The EDS peak values showed that the presence of SiC particles in the SZ was clearly confirmed.

### 3.3. Mechanical Properties of the AZ31B/SiC Composite Joints

Ineffectual tool rotation speed and tool traveling feed led to a good agreement between the FSW joints and produced promising mechanical properties, such as ultimate tensile strength, hardness, and remarkable fracture surface. The SZ mechanical properties were very sensitive to the following: (i) grain refinement in the weld nugget zone, (ii) pinning effect of the particles, and (iii) formation of clusters in the SZ [16,20,26]. The assessment values of the nano-composite joints are summarized in the Table 4.

#### 3.3.1. Tensile Properties 

In the FSW process, the addition of nanoparticles had an important effect on the tensile strength and percent elongation values (POE) [7]. In particular, Gopalakrishnan et al. [27] found the tensile strength value decreased up to 3% with the addition of TiC particles in the aluminum matrix composite (Al-TiC) when compared to cast aluminum strength. When the percentage of the addition of TiC went beyond the limit (up to 7% addition of TiC), the ultimate tensile strength (UTS) values were found to increase steadily. Similarly, the UTS value increased with the increase in the percentage addition of SiC particles within the SZ up to the maximum limit of specimen V12. The continuous addition of SiC nanoparticles in the SZ offered more resistance to plastic deformation during a tensile test. Additionally, the stress (load) transformation between the matrix and harder SiC reinforcement gave more strength to the AZ31B/SiC composite joints. According to the hall patch relationship, the pinning effect of the reinforcement particles created new, localized fine grains in the weld nugget and resulted in offering the highest tensile strength [19,28]. This behavior was in good agreement with the specimens V0 to V12, which showed continuous improvement of UTS and POE values, respectively, as represented in Table 4. The maximum UTS value of specimen V12 was 235.81 MPa, which was 21% higher than that of the specimen without SiC. Also, the concurrent improvement was observed in the POE and hardness values of specimens V0 to V12.

**Figure 12:**
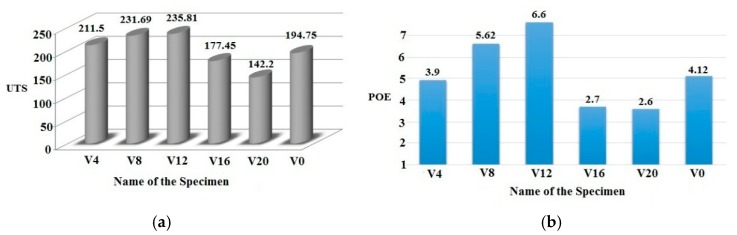
Comparative analysis of AZ31B/SiC nanocomposite joints on (**a**) ultimate tensile strength (UTS); (**b**) percentage of elongation (POE).

However, in the case of specimens V16 and V20, the above-stated trend was non-linear. Upon further addition of SiC reinforcement in the SZ, the UTS and the POE also decreased by a considerable amount compared to the specimens stated above, because while adding SiC of particles more than 12%, the ductile properties decreased due to the lower level of dispersion and agglomeration of the SiC nanoparticles. From the SEM micrographs (Figure 10), the cluster formations in the specimens V4, V8, and V12 revealed a negligible difference due to a fine dispersion of the SiC particles and a good bonding between the SiC and the AZ31B magnesium alloy in the SZ. Specimens V16 and V20 showed bigger sized clusters due to poor stirring action of the rotating tool and reduction in interparticle spacing between the SiC particles or reduction in interfacial bonding between the SiC particle and the magnesium matrix. Finally, the non-uniform dispersion of the SiC particles and cluster formations deteriorated the UTS and the POE values [29]. Figure 12 shows the comparative analysis of the UTS and the POE values in AZ31B/SiC nano-composite joints.

#### 3.3.2. Fractography Study

**Figure 13:**
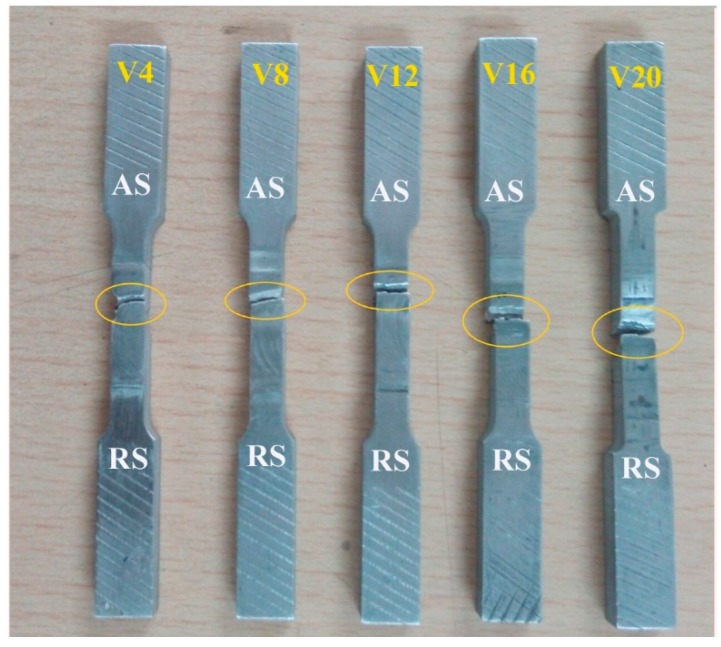
Typical fractured regions of the tensile test specimens.

Figure 13 presents the fractured region of the FSW of SiC reinforced AZ31B magnesium matrix nano-composite with various volume fractioned samples. According to the figure, tensile samples of V4, V8, and V12 failed between the thermo-mechanically affected zone (TMAZ) and the HAZ on the advancing side. These were attributed to the following reasons: (i) dislocation density increased in TMAZ due to inadequate plastic deformation, (ii) tensile stress was continuously applied in these zones until it reached the level of crack nucleation, and (iii) interfacial bonding between the SiC nanoparticles and the magnesium matrix in the SZ restricted the formation of a crack. This meant that the lack of plasticization was the factor responsible for producing larger grain boundaries within the TMAZ and the HAZ. This was seemingly due to the poor wettability of the SiC and magnesium matrix phases, which led to a weak cohesion of these phases. These results show parallel conformity with those of Thangarasu et al. [16], who observed the fracture away from the SZ of TiC reinforced FSP of AA6082/TiC composite joints. On the contrary, samples V16 and V20 failed in the SZ due to poor wettability between the reinforcement and the matrix alloy. It was also in excellent conformity with the tensile values in ascending order followed by samples V4, V8, and V12. 

**Figure 14:**
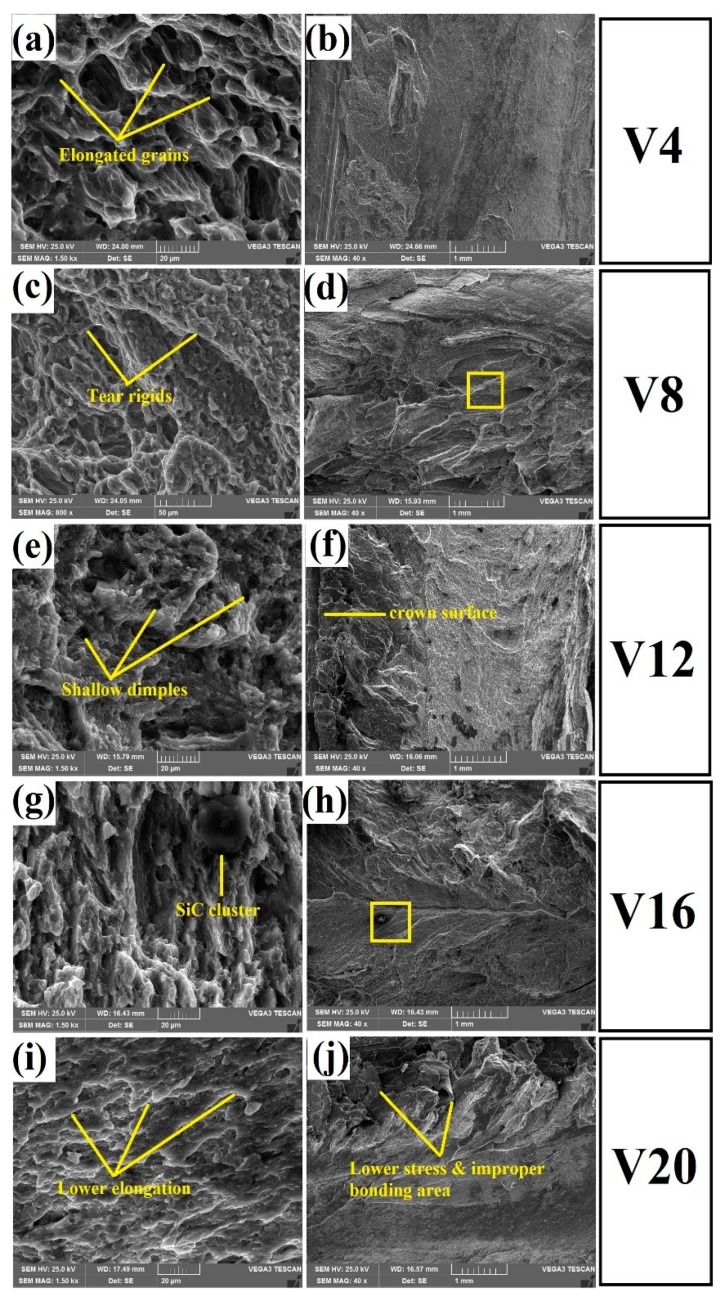
SEM fractography of the tensile specimens with macrographs of (**a**,**b**) V4—high and low magnification; (**c**,**d**) V8—high and low magnification; (**e**,**f**) V12—high and low magnification; (**g**,**h**) V16—high and low magnification; (**I**,**j**) V20—high and low magnification.

Figure 14 demonstrates the high magnification SEM micrographs of the fractured surface along with the macro fractographs. Cohesive bonding between the accumulated SiC and the magnesium matrix reduced the strength and elongation, as well as adhesive bonding between the reinforcements and substrates, and improved the strength and elongation of the FSW joints [6]. However, specimens V4, V8, and V12 revealed good bonding between the SiC nano-particles and the magnesium matrix due to adequate thermal heat generation, and the fractured surfaces indicated a ductile manner. The fractured surface showed a large number of dimples and voids, as shown in Figure 14a,c,e. Indeed, the presence of dimples and voids directly influenced the elongations of the specimen during tensile testing [30,31,32]. Some of the tearrigids and shallow dimples were observed in the V12 specimen, which showed moderate elongation, as shown in Figure 14e. Specimens V16 and V20 failed in the SZ, and the fracture surfaces indicated their brittle nature [33], which was the reason for the lower level of dispersion and agglomeration of SiC nanoparticles in the SZ, as mentioned earlier. Although specimens V16 and V20 fractured from the SiC nanoparticles’ accumulated areas, it was evident that a bigger sized SiC cluster was indicated in the fractured surface of specimen V16, as shown in Figure 14g. This similarity was observed in the fabrication of the AA7075/SiC nano-composite joints [7] and also a good agreement with pure copper joints reinforced with SiC particles [12]. The V20 specimen, however, showed no major elongation. A cleavage type fracture mode was observed in the fracture location, and this produced low elongation.

#### 3.3.3. Microhardness Study 

**Figure 15:**
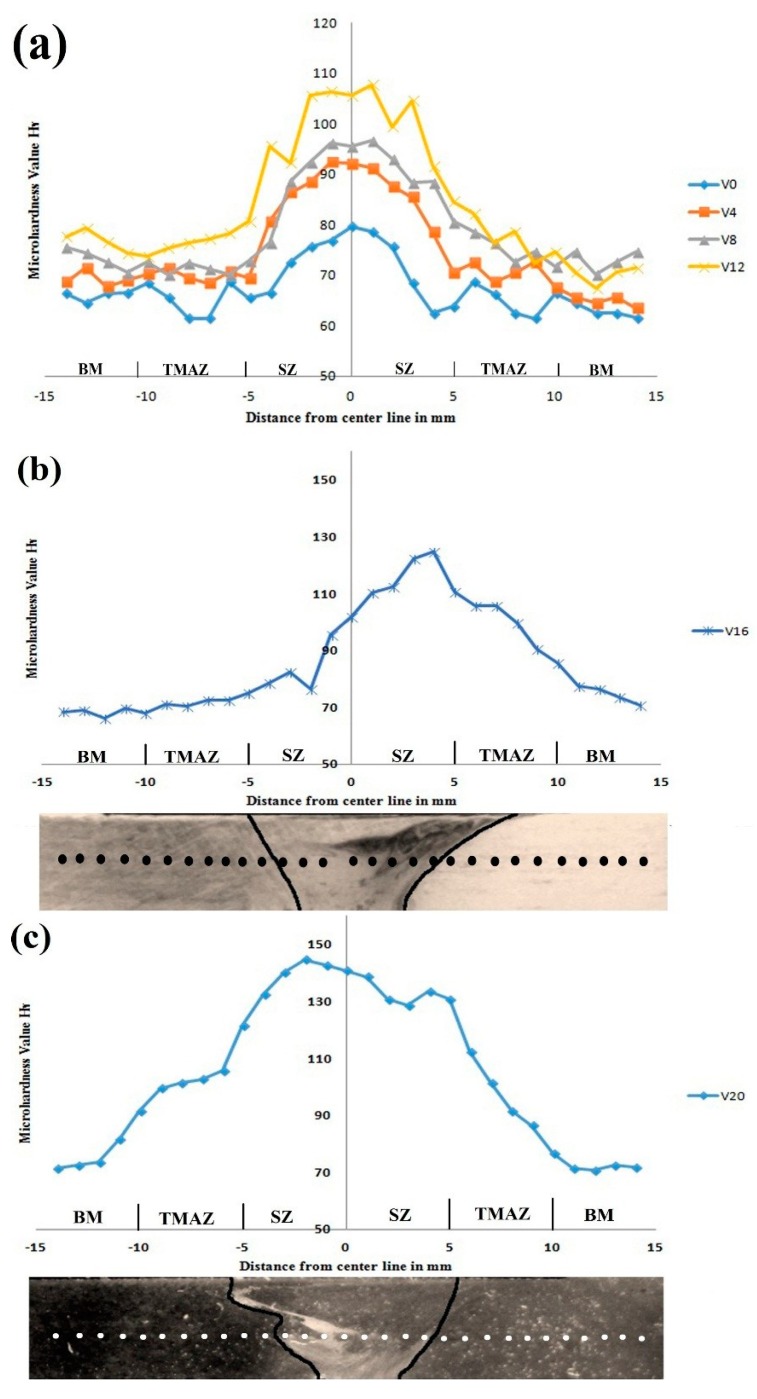
Microhardness distributions of AZ31B/SiC composite welded joints across the center line (**a**) V0 to V12 (**b**) V16 (**c**) V20.

The hardness distribution of various volume fractioned AZ31B/SiC composite welded joints is shown in Figure 15. Generally, the responsible factors of hardness are fine grain structure, sufficient dispersion of reinforcement in the matrix, and variable thermal expansion coefficient between the hard reinforcement and the matrix alloy [34]. The ebb and flow shaped profile clearly confirmed the presence of SiC rich and SiC free regions in the weld nugget zones. Nevertheless, a low hardness value was observed in the particle free field, and high hardness value was observed in the particle rich field [19]. The microhardness values were observed from the specimens across the center line of the welded joints, and as the benchmark for comparison, the average microhardness value of the composite joints is shown in Table 4. It could be inferred that the average microhardness value in the SZ of AZ31B with dispersed SiC nanoparticles was higher than that of the zero volume fractioned counterparts, irrespective of the volume fractions. On the other hand, the average microhardness value of specimens V4, V8, and V12 increased while increasing the SiC reinforcement, according to the percentage addition, which was seemingly due to the following reasons: (a) the uniform distribution of SiC particles and grain size refinement (as per the Hall-Petch theory) in the SZ enhanced the microhardness values [15]; (b) according to the Orowan theory, reinforcements and matrix alloy exhibit different coefficients of thermal expansion, leading to the improvement of the dislocation density due to the formation of residual stresses during cooling time. Hence, the volume fraction increased the number of dislocations in the AZ31B alloy due to more SiC particles [16]; (c) an interparticle spacing decreased in the SZ due to the decrease in the ASC, thus there was an increase in the microhardness value. On the contrary, at a constant volume fraction condition, the interparticle spacing increased in the SZ due to increases in the ASC, which subsequently decreased the microhardness value [21]. Insufficient heat generation during the FSW process led to the production of clusters and the accumulation of reinforcements (non-uniform dispersion), which increased the microhardness value. Figure 15b represents the average microhardness value of specimen V16, which increased due to the non-homogeneous distribution of TMAZ and SZ. Hence, the hardness distribution in the TMAZ reached the maximum value. Figure 15c shows the average microhardness value of the V20 specimen with the highest microhardness value compared to the other welded joints. In other words, the SiC particles accumulated at the bottom of the welded sample, and it therefore had a higher average microhardness value. Moreover, the hardness profile of the V4, V8, and V12 specimens produced a uniform dispersion of nanoparticles as well as fine grain sizes within the SZ compared to the V16 and V20 specimens.

## 4. Conclusions

AZ31B magnesium alloy was successfully reinforced with SiC nanoparticles and FSW conducted with five different SZ volume fractions, and its significant effect on the macrostructure, microstructure, and mechanical behavior of composite joints was studied. From this research, the following findings were made:

- The absence of defect-free composite welded joints was achieved at the constant tool rotational speed of 1250 rpm and tool traveling speed of 25 mm/min, irrespective of the volume fractions.
- The areas of the surface composite of welded joints of specimens V4 to V20 were decreased from 67.45 mm^2^ to 42.35 mm^2^. It was concluded that the volume fractions of friction stir welded composite joints were indirectly proportional to the ASC.
- Specimens V4, V8, and V12 were obtained with homogeneous particle dispersion within the SZ in the given welding condition. On the contrary, specimens V16 and V20 were observed to have severe SiC nano-particle accumulations as well as cluster formations due to insufficient heat generation around the tool pin profile.
- Heat transfer was decreased between the transition zones while increasing the volume fractions as well as increasing the density of SiC nanoparticles in the SZ.
- SiC nanoparticles influenced both the UTS and the POE values of the composite welded joints. The UTS and the POE values increased with the increase in the volume fractions of joints from V4 to V12 and decreased when there was further increase in volume fractions up to specimen V20 due to inadequate material mixing with the reinforcement.
- The fractography of the failed samples showed that the brittle fracture mode was observed in the SiC particles accumulated joints, which loosened the bonding structure within the SZ (specimens V16 and V20), whereas others followed the ductile fracture mode and failed in between the TMAZ and the HAZ (specimens V4, V8, and V12).
- Uniform hardness distribution in specimens V4, V8, and V12 maintained a good conformity with the Hall-Patch relationship and dispersion of the SiC particles. Other specimens were not up to that level.

## Figures and Tables

**Table 1:** Composition of AZ31B magnesium alloy (wt. %).

Al	Zn	Mn	Si	Cu	Ca	Ni	Fe	Other	Mg
3.12	0.68	0.23	0.011	0.00021	0.031	0.0048	0.0003	0.19	Balance

**Table 2:** Properties of the AZ31B magnesium alloy.

Yield Strength (MPa)	Ultimate Tensile Strength (MPa)	Elongation in (%)	Hardness at 0.025 Kg Load (HV)
124	234	14	86

**Table 3:** Fraction stir welding (FSW) process parameters used in this study.

Control Parameters	Values
Tool rotational speed, N (rpm)	1250 rpm
Tool travel rate, F (mm/min)	25 mm/min
Volume percentage V (%)	4 (V4), 8 (V8), 12 (V12), 16 (V16), 20 (V20)
Groove width (W)	0.3, 0.6, 0.9, 1.2, 1.5
D/d ratio and Pin height in mm	3 and 5.7 mm
Tool Pin profile	Cylindrical threaded tool pin profile
Tool material	H13 tool steel

**Table 4:** Assessment values of AZ31B/SiC volume fractioned samples and the base material.

Specimen Name	V4	V8	V12	V16	V20	V0
Theoretical Volume Percentage	4%	8%	12%	16%	20%	0%
Actual Volume Percentage	2%	4.5%	7.47%	11%	14.40%	-
Average Microhardness (Hv)	75	79	84	93	112	68
Ultimate Tensile Strength (MPa)	211.5	231	235.81	177	142.2	195
Percentage of Elongation (POE)	3.9	5.62	6.6	2.7	2.6	4.12

## Abbreviations

FSW	Friction Stir Welding
MMC	Magnesium Matrix Composite
SZ	Stir Zone
HAZ	Heat Affected Zone
TMAZ	Thermo Mechanically Affected Zone
UTS	Ultimate Tensile Strength
POE	Percentage of Elongation
